# Are Dichorionic Twin Pregnancies Resulting from In Vitro Fertilization Different from Spontaneous Dichorionic Twin Pregnancies? A Retrospective Cohort Study

**DOI:** 10.3390/jcm14228000

**Published:** 2025-11-11

**Authors:** Ahmet Zeki Nessar, Şebnem Karagün, Fikriye Işıl Adıgüzel, Şule Gül Aydın, Serdar Aykut, Aslıhan Kurt, Süleyman Cansun Demir, Mete Sucu, İsmail Cüneyt Evrüke

**Affiliations:** 1 Division of Perinatology, Department of Obstetrics and Gynecology, Osmaniye State Hospital, Osmaniye 80000, Turkey; 2 Department of Perinatology, Adana City Hospital, University of Health Sciences, Adana 01230, Turkey; karagunsebnem@gmail.com; 3 Medical Park Adana Hospital, Adana 01060, Turkey; aze_isil@hotmail.com; 4 Department of Obstetrics and Gynecology, Adana City Hospital, University of Health Sciences, Adana 01230, Turkey; sulegulaydin@gmail.com; 5 Division of Perinatology, Department of Obstetrics and Gynecology, Mardin Training and Research Hospital, Mardin 47100, Turkey; drserdaraykut@gmail.com; 6 Department of Perinatology, Gaziantep City Hospital, University of Health Sciences, Gaziantep 27060, Turkey; aslihanzeylikurt@gmail.com; 7 Division of Perinatology, Department of Obstetrics and Gynecology, Çukurova Univercity, Adana 01330, Turkey; cansundemir@gmail.com (S.C.D.); metesucu@yahoo.com (M.S.); cuneytevruke@gmail.com (İ.C.E.)

**Keywords:** in vitro fertilization, multifetal pregnancy, perinatal outcomes

## Abstract

**Background**: We aimed to compare the perinatal outcomes of dichorionic/diamniotic twin (DC/DA) pregnancies resulting from in vitro fertilization (IVF) with those resulting from spontaneous DC/DA pregnancies. **Methods**: The study group included 99 women with DC/DA pregnancies resulting from IVF, and the control group included 92 women with spontaneous DC/DA pregnancies. Maternal demographic characteristics (age, parity, and gravidity), pre-existing conditions (chronic hypertension and pregestational diabetes mellitus), and obstetric history were recorded. Pregnancy outcomes included gestational age at delivery, number of fetuses, and mode of delivery. The antepartum complications that we evaluated include first- and second-trimester bleeding, placenta previa, preterm birth, fetal growth restriction (FGR), oligohydramnios, and tocolytic use. The obstetric complications that we assessed include prematurity, twin-to-twin transfusion syndrome (TTTS), and hydrops fetalis. Additionally, neonatal data such as 1st minute and 5th minute Apgar scores, birth weight, neonatal intensive care unit (NICU) admission, presence of congenital anomalies, and neonatal death were recorded, and comparisons were made between the groups. **Results**: Women in the IVF group were older (34.7 ± 6.9 vs. 32.3 ± 6.1 years, *p* = 0.03) and more frequently primiparous (73.7% vs. 37.0%, *p* < 0.001). The mean gestational age at delivery was slightly lower in the IVF group, though this was not statistically significant (34.3 ± 3.5 vs. 35.1 ± 2.5 weeks, *p* = 0.101). Cesarean delivery was common in both groups, with comparable overall rates (90.9% vs. 94.6%, *p* = 0.411), but emergency cesarean section occurred more frequently in IVF pregnancies (81.8% vs. 55.8%, *p* = 0.001). No significant differences were found regarding chronic hypertension or pregestational diabetes. However, several differences were demonstrated in terms of obstetric complications. For example, preterm births and fetal growth restriction (FGR) were significantly more frequent in IVF pregnancies (59.8% vs. 30.4%, *p* < 0.001), and tocolytic use was also more frequent (56.6% vs. 29.7%, *p* < 0.001). No significant differences were observed in terms of placenta previa, oligohydramnios, TTTS, hydrops fetalis, and neonatal outcomes. The logistic regression analysis revealed that IVF pregnancies were associated with an increased risk of preterm birth: OR 3.45, 95% CI 1.85–6.78 (*p* < 0.001); the risk of FGR was also higher in IVF pregnancies: OR 2.11, 95% CI 1.02–4.37 (*p* = 0.015). However, tocolytic use was not significantly associated with IVF: OR 1.49, 95% CI 0.50–4.44 (*p* = 0.471). **Conclusions:** Although DC/DA pregnancies conceived through IVF have a higher risk of preterm birth, fetal growth restriction, and greater use of tocolytic agents than spontaneous DC/DA pregnancies, their neonatal outcomes are similar.

## 1. Introduction

In parallel with the rapid advancements in assisted reproductive technologies (ARTs) that have been made over the last 30 years, the share of babies born as a result of ART pregnancies in developed countries has reached 4% of all live births [1]. ART includes techniques such as in vitro fertilization (IVF), intracytoplasmic sperm injection (ICSI), embryo cryopreservation, and preimplantation genetic diagnosis. An IVF cycle consists of several stages; these can be summarized as controlled ovarian hyperstimulation, oocyte retrieval, oocyte fertilization with sperm, embryo culture, and, finally, embryo transfer into the uterus [2]. For a long time, many studies have demonstrated an increased risk of adverse pregnancy complications in IVF pregnancies. Complications with an increased risk of IVF include placental abruption, pregnancy loss after 24 weeks’ gestation, preeclampsia, gestational diabetes and hypertension, placenta previa, and an increased rate of cesarean section [3]. There are also studies indicating that the risk of small-for-gestational-age (SGA) newborns increases in women who conceive through IVF [4]. Regarding pregnancy complications, maternal age, smoking, endometriosis, multifetal pregnancy, obesity, and history of ovarian hyperstimulation in early pregnancy (OHSS) are the main prognostic factors [5]. In addition, in IVF pregnancies that resulted in miscarriage, the resistance measured in uterine artery Doppler at the 6th week of pregnancy was shown to be lower compared with that in ongoing pregnancies [6].

In the early years of IVF treatment, multiple embryo transfers were common to increase pregnancy rates. Over time, with advancements in technology in ART practices and increased implantation rates, multiple embryo transfers began to result in an increase in multifetal pregnancies. While measures have been taken in many countries to prevent this, the rate of multifetal pregnancies in IVF pregnancies remains high worldwide [7]. It has been suggested that the frozen–thawed embryo transfer (FET) strategy may reduce the rate of multifetal pregnancies [8]. Multifetal pregnancies are high-risk pregnancies that can be complicated by premature birth, low birth weight, preeclampsia, anemia, postpartum hemorrhage, intrauterine growth restriction (IUGR), neonatal morbidity, and high neonatal and infant mortality rates. The risk of perinatal death increases fourfold for twins and sixfold for triplets [9].

There are many studies comparing IVF pregnancies with spontaneous pregnancies. It has been determined that older women who conceive through IVF experience more obstetric complications than those who conceive spontaneously [10]. The causes of recurrent miscarriage in spontaneous and IVF pregnancies have been shown to be similar [11]. A study comparing spontaneous and IVF monochorionic diamniotic twin pregnancies found no difference in perinatal outcomes [12]. We designed this study to investigate whether spontaneous and IVF multifetal pregnancies differ in terms of obstetric complications.

## 2. Materials and Methods

**Figure 1:**
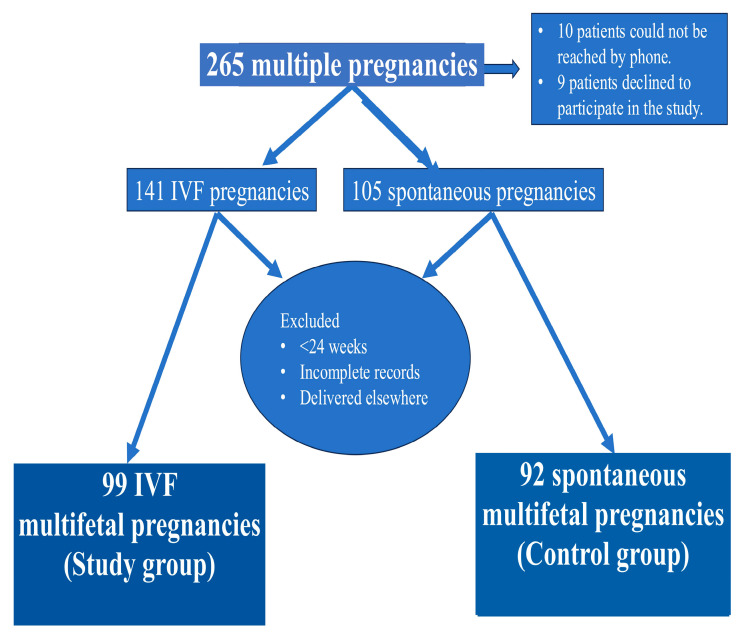
Flowchart of study participants.

Between January 2018 and December 2023, the digital medical records and physical patient files of women with dichorionic/diamniotic twin (DC/DA) pregnancies who were followed up and treated at the Obstetrics and Gynecology Clinic of Çukurova University Faculty of Medicine Hospital, a tertiary healthcare center, were reviewed. Two hundred and sixty-five DC/DA pregnancies were identified. Patients were contacted by phone, invited to the hospital, and informed consent forms were obtained after delivery. Ten patients could not be reached by phone, and nine patients declined to participate in the study; therefore, the study continued with 246 patients. While 141 of these women had multifetal pregnancies as a result of IVF treatment, the remaining 105 women had spontaneous multifetal pregnancies. Those whose pregnancies did not continue beyond 24 weeks, those with incomplete records, and those who gave birth at another facility were excluded from the study. Consequently, 99 women were included in the IVF DC/DA pregnancy group (study group) and 92 women were included in the spontaneous DC/DA pregnancy group (control group) (Figure 1).

The eligibility criteria were confirmed to be multifetal gestation (≥2 fetuses), delivery at ≥24 weeks of gestation, and availability of complete maternal and neonatal data. The exclusion criteria included pregnancies complicated by major congenital malformations, selective fetal reduction, intrauterine demise before 24 weeks, or incomplete medical records. Monochorionic multifetal pregnancies were excluded from both groups because monochorionic multifetal pregnancies have unique complications.

Maternal demographic characteristics (age, parity, and gravidity), pre-existing conditions (chronic hypertension and pregestational diabetes mellitus), and obstetric history were extracted from the patients’ files. Pregnant women over 35 years of age are considered to be of advanced maternal age; accordingly, the patients were divided into two groups: those under 35 years of age and those over 35 years of age [13]. Pregnancy outcomes included gestational age at delivery, number of fetuses, and mode of delivery. The antepartum complications that we evaluated include first- and second-trimester bleeding, placenta previa, preterm birth, fetal growth restriction (FGR), oligohydramnios, and tocolytic use. The fetal complications that we assessed include prematurity, twin-to-twin transfusion syndrome (TTTS), hydrops fetalis, and neonatal death.

For uncomplicated dichorionic/diamniotic twin pregnancies, we planned delivery at 38 + 0 to 38 + 6 weeks of gestation, in agreement with the recommendations of the American College of Obstetricians and Gynecologists [14]. Preterm birth was defined as delivery before 37 completed weeks of gestation [15]. Fetal growth restriction (FGR) was defined as an estimated fetal weight below the 10th percentile for gestational age [16]. Fetal hydrops was defined as fluid accumulation in two or more body cavities. The TTTS diagnosis was made according to Quintero staging [17]. Oligohydramnios was defined as an amniotic fluid index (AFI) of 5 cm or less [18]. In our center, nifedipine is used for tocolysis, and tocolysis is used in the setting of preterm labor. Preterm birth is considered to be delivery before 37 weeks of gestation and after 20 weeks. To diagnose preterm labor, continued contractions must happen during the gestational age range mentioned previously to produce cervical changes; continued contractions are defined as greater than or equal to 4 contractions over 20 min [19]. Although the pregnant women in the IVF group used progesterone in the first trimester, there was no routine use in those who conceived spontaneously.

All statistical analyses were performed using IBM SPSS Statistics, version 26 (IBM Corp., Armonk, NY, USA). Continuous variables were expressed as mean ± standard deviation (SD) or median with interquartile range (IQR), as appropriate, and compared using an independent-samples *t*-test or Mann–Whitney U test. Categorical variables were summarized as numbers and percentages and compared using the Chi-square test or Fisher’s exact test. To identify the factors independently associated with IVF-related multifetal gestation, a binary logistic regression analysis was performed. Odds ratios (ORs) with 95% confidence intervals (CIs) were calculated, and statistical significance was defined as *p* < 0.05.

## 3. Results

There were no statistically significant differences between the groups in terms of age (*p* = 0.362). Primiparity was more frequent in IVF pregnancies, at 73.7%, compared with 63.0% in the spontaneous group (*p* < 0.001). Conversely, multiparity was more common in spontaneous pregnancies, at 37.0% versus 26.3% (*p* < 0.001).

The mean gestational age at delivery was 33.2 ± 3.3 weeks in the IVF group and 35.1 ± 2.5 weeks in the spontaneous group (*p* < 0.001). Cesarean section was the predominant delivery mode in both groups, at 90.9% in IVF pregnancies and 94.6% in spontaneous pregnancies (*p* = 0.411). Emergency cesarean section was significantly more frequent in IVF pregnancies, at 81.8%, compared with 55.8% in spontaneous pregnancies (*p* = 0.001). There were no significant differences between the groups regarding chronic hypertension (*p* = 0.332) or pregestational diabetes mellitus (*p* = 0.167) (Table 1).

The rate of preterm birth was higher in IVF pregnancies, at 59.8%, compared with 30.4% in spontaneous pregnancies (*p* < 0.001). FGR was also more common in the IVF group, at 31.3%, versus 19.5% in the spontaneous group (*p* = 0.020). Oligohydramnios, placenta previa, and first-/second-trimester bleeding rates were similar in both groups. TTTS occurred in 16.3% of spontaneous and 12.4% of IVF pregnancies (*p* = 0.084), while hydrops fetalis was reported in 1.1% of spontaneous and 0% of IVF pregnancies (*p* = 0.487). The rate of tocolytic use was higher in IVF pregnancies, at 56.6%, versus 29.7% in spontaneous pregnancies (*p* < 0.001) (Table 2).

The median Apgar score at 1 min was seven in both groups (*p* = 0.213). At 5 min, the median Apgar score was nine in the IVF group and eight in the spontaneous group (*p* = 0.609). The median birth weight was 2200 g in IVF babies and 2565 g in spontaneously conceived babies (*p* < 0.001). NICU admission occurred in 59.6% of IVF and 53.0% of spontaneous newborns (*p* = 0.441). The incidence of fetal anomalies did not differ significantly between groups (*p* = 0.355). Neonatal mortality was 3.1% in the IVF group and 1.1% in the spontaneous group (*p* = 0.622) (Table 3).

The logistic regression analysis revealed that IVF pregnancies were associated with an increased risk of preterm birth: OR 3.45, 95% CI 1.85–6.78 (*p* < 0.001); the risk of FGR was also higher in IVF pregnancies: OR 2.11, 95% CI 1.02–4.37 (*p* = 0.015). However, tocolytic use was not significantly associated with IVF: OR 1.49, 95% CI 0.50–4.44 (*p* = 0.471) (Table 4).

## 4. Discussion

In our study, we observed that women with DC/DA pregnancies resulting from IVF treatment were older, had fewer pregnancies, and had fewer children compared with those with spontaneous DC/DA pregnancies. We observed a higher rate of preterm birth and more frequent use of tocolytics in the IVF group. The diversity in the etiology of preterm birth complicates the selection of treatment options to prevent this condition. Therefore, tocolytic therapy is administered in our clinic to facilitate antenatal steroid therapy and patient transfer; we believe this may have contributed to the high preterm birth rate [19]. Despite this, the fetal complications in those who conceived through IVF and those with spontaneous pregnancies were similar.

Assisted reproductive techniques, particularly IVF, have been widely used in the treatment of infertile or subfertile couples worldwide for over 40 years, with the birth of the first IVF baby in 1978 [20]. While IVF treatment offers great hope for couples seeking children, there has been debate from the start about whether pregnancies achieved through this treatment differ from naturally occurring pregnancies. Studies have shown that IVF pregnancies have a higher risk of spontaneous preterm birth, higher bile acid levels, and higher rates of gestational diabetes, hypertension, multifetal pregnancy, and cesarean section [10,21,22]. Additionally, the rate of preeclampsia has been found to be higher in IVF pregnancies than in spontaneous pregnancies [23]. Furthermore, some researchers have also found that placental and umbilical cord anomalies are common in IVF pregnancies and that, as a result, there is a higher risk of adverse perinatal outcomes [24]. In our study, there were no statistical differences between the groups in terms of preeclampsia. We do not recommend routine aspirin use in twin pregnancies in our country.

Considering all these findings, we thought it would be useful to compare spontaneous multifetal pregnancies followed up and treated in our clinic with multifetal pregnancies resulting from IVF. In our study, the average age of the women in the IVF group was higher than that of the women in the spontaneous group. A higher average age of those who conceived as a result of IVF treatment has been observed in many studies. Lin et al. compared spontaneous and IVF dichorionic–diamniotic twin pregnancies in terms of preterm birth and found that women in the IVF group were older [25]. Similarly, in their study comparing monochorionic diamniotic twin pregnancies, Roero et al. observed that the average age of women in the IVF group was higher [12].

The parity and number of children in the study group were lower than in the control group. This finding is consistent with the findings of multiple previous studies. In their study examining preeclampsia rates in twin pregnancies, Okby et al. found that nulliparity was more common in women in the IVF group [26].

We found that FGR was higher in the study group. Furthermore, birth weight was significantly lower in the IVF group. There are some studies showing that women who conceive via ART techniques do not have a higher risk of FGR than those who conceive spontaneously [27]. However, in a recent study, Lang et al. determined that babies of women conceived through IVF had lower birth weights than those conceived naturally [10].

Our study found that the IVF group had a higher rate of preterm birth and required more tocolytic use. Saccone et al. found that IVF twin pregnancies had a higher rate of spontaneous preterm birth and shorter mid-trimester cervical lengths compared with spontaneous twin pregnancies [28]. Li et al. compared IVF and spontaneous multifetal pregnancies and stated that IVF pregnancies had a higher risk of preterm birth [25]. Similarly, some other studies show that the risk of preterm birth and prematurity is high in IVF pregnancies [25,29].

The incidence of previa with ART is several-fold higher than that with non-ART conceptions, with one systematic review providing an odds ratio of 3.76 [30]. However, in our study, there were no significant differences between the two groups in terms of placenta previa.

We observed a higher rate of emergency cesarean sections in the IVF group, likely due to the increased need for emergency cesarean sections as a result of the increased risk of preterm labor. Studies have also indicated an increased risk of cesarean sections in multifetal pregnancies resulting from IVF [31]. In their study, Duy et al. stated that the cesarean rate is high in IVF twin pregnancies [29].

When looking at neonatal outcomes, the results in both groups in our study were similar; Levi Setti et al. also obtained similar results [32].

The primary limitations of this study include its retrospective nature, limited sample size, and single-center nature. Furthermore, the IVF protocol used was performed at various centers, and we have limited information about IVF cycles outside our center. In our country, twin pregnancies are an indication of elective cesarean section. If the patient requests a cesarean section, it is performed. This is a limitation that led to the high cesarean rate in our study. Finally, due to the retrospective nature of this study, we were unable to include first-trimester pregnancy losses.

## 5. Conclusions

DC/DA pregnancies resulting from IVF carry greater risks than spontaneous DC/DA pregnancies. In particular, preterm labor and prematurity are more common in IVF pregnancies than in spontaneous pregnancies. Therefore, we believe that these women require closer and more intensive monitoring. However, we believe that IVF pregnancies are no different from spontaneous DC/DA pregnancies in terms of neonatal complications.

## Figures and Tables

**Table 1:** Maternal demographics and delivery characteristics of multifetal gestations following IVF or spontaneous conception.

Variable	IVF (*n* = 99)	Spontaneous (*n* = 92)	*p*-Value
Age (years)			0.362
<35	56 (56.6%)	58 (63%)	
≥35	43 (43.4%)	34 (37%)	
Nulliparous	73 (73.7%)	34 (37%)	<0.001
Parous	58 (63%)	26 (26.3%)	<0.001
Gestational age at delivery (weeks) mean ± S.D.	33.24 ± 3.28	35.10 ± 2.50	<0.001
Delivery type			0.411
Vaginal delivery	9 (9.1%)	5 (5.4%)	
Cesarean section	90 (90.9%)	87 (94.6%)	
Emergency cesarean section	81 (81.8%)	55 (55.8%)	0.001
Chronic hypertension	7 (7.9%)	11 (12.5%)	0.332
Pregestational DM	10 (10.1%)	4 (4.3%)	0.167

Abbreviations: IVF: in vitro fertilization; S.D.: standard deviation; DM: diabetes mellitus.

**Table 2:** Antepartum complications among multifetal gestations following in vitro fertilization (IVF), ovarian stimulation, or spontaneous conception.

Variable	IVF (*n* = 99)	Spontaneous (*n* = 92)	*p*-Value
First- and second-trimester bleeding	47 (48.5%)	44 (47.8%)	0.524
Preeclampsia			0.308
(+)	7 (7.1%)	11 (11.9%)	
(−)	92 (92.3%)	81 (88.1%)	
Placenta previa	5 (5.2%)	3 (3.3%)	0.390
Preterm birth	58 (59.8%)	28 (30.4%)	<0.001
FGR	31 (31.3%)	18 (19.5%)	0.020
Oligohydramnios	12 (12.4%)	15 (16.3%)	0.534
TTTS	12 (12.4%)	21 (22.8%)	0.084
Hydrops fetalis	0 (0%)	1 (1.1%)	0.487
Tocolytic use	56 (56.6%)	27 (29.7%)	<0.001

Abbreviations: IVF: in vitro fertilization; FGR: fetal growth restriction; TTTS: twin-to-twin transfusion syndrome.

**Table 3:** Neonatal complications among multifetal gestations following in vitro fertilization (IVF), ovarian stimulation, or spontaneous conception.

Variable	IVF Babies (*n* = 204)	Spontaneous Babies (*n* = 185)	*p*-Value
Apgar 1. min. median (IQR)	7 (3)	7 (2)	0.213
Apgar 5. min. median (IQR)	9 (1)	8 (1)	0.609
Birth weight (g.) median (IQR)	2200 (675)	2565 (1356)	<0.001
Fetal anomaly *n* (%)			0.355
Cardiac anomaly	3 (1%)	0 (0%)	
CNS anomaly	2 (0.9%)	1 (0.5%)	
GIS anomaly	1 (0.4%)	0 (0%)	
Urinary anomaly	1 (0.4%)	0 (0%)	
NIUC	116 (59.6%)	98 (53%)	0.441
Neonatal death	3 (3.1%)	1 (1.1%)	0.622

Abbreviations: IVF: in vitro fertilization; CNS: central nervous system; GIS: gastrointestinal system; NIUC: neonatal intensive care unit.

**Table 4:** Multiple logistic regression analysis of factors related to IVF.

Variable	OR (95% CI)	*p*-Value
Age	0.965 (0.500–1.859)	0.914
Preeclampsia	1.511 (0.484–4.714)	0.477
FGR	2.119 (1.026–4.377)	0.043
Tocolytic use	1.493 (0.502–4.441)	0.471
Preterm birth	3.457 (1.854–6.786)	<0.001

Abbreviations: IVF: in vitro fertilization, OR: odds ratio, FGR: fetal growth restriction.

## Data Availability

Derived data supporting the findings of this study are available from the corresponding author.
